# 2147: Relationships between medical complexity factors and medication confidence and adherence among older Singaporean adults

**DOI:** 10.1017/cts.2017.247

**Published:** 2018-05-10

**Authors:** Stacey Ying Guo, Heather Whitson, Truls Ostbye, Alison Luciano, Rahul Malhotra

**Affiliations:** Duke University, Durham, NC, USA

## Abstract

OBJECTIVES/SPECIFIC AIMS: To investigate whether medical complexity (indicated by multiple providers or healthcare visits) is associated with lower levels of confidence in medication use and lower medication adherence METHODS/STUDY POPULATION: Data on socio-demographics, health encounters, health status, and health attitudes and behaviors from a nationally representative sample of 1575 older Singaporean adults were utilized. The association of medical complexity factors with self-reported medication confidence and adherence was analyzed using logistic regression analysis controlling for age, gender, ethnicity, education, and number of health conditions. RESULTS/ANTICIPATED RESULTS: The survey had a 60% response rate. The mean age of respondents was 72, and 42% were male. We found no significant association between number of visits and either confidence about usage (OR=1.07, 95% CI 0.95–1.20) or medication adherence (OR=1.01, 95% CI 0.90–1.13). We similarly found no significant association between number of providers and either confidence about usage (OR=1.03, 95% CI 0.90–1.18) or medication adherence (OR=1.05, 95% CI 0.93–1.20). Lower confidence about medication use was less likely among males (OR=0.60, 95% CI 0.44–0.80), those with more education (OR=0.29, 95% CI 0.20–0.42) or more comorbidities (OR=0.89, 95% CI 0.82–0.96) and more likely with increasing age (OR=1.06, 95% CI 1.04–1.08). Nonadherence was more likely among Indians (OR=1.62, 95% CI 1.06–2.48) and those with more comorbidities (OR=1.10, 95% CI 1.02–1.18). DISCUSSION/SIGNIFICANCE OF IMPACT: Having more healthcare visits or providers were not independent correlates of lower medication confidence or adherence. Seniors with less education may benefit from interventions to improve confidence about medication use. Participants with more comorbidities expressed greater confidence but admitted to lower adherence. The role of other potential contributors to nonadherence in complex patients (eg, cost and access, patient preference, competing demands) should be evaluated next.

